# HIV, Other Blood-Borne Viruses and Sexually Transmitted Infections amongst Expatriates and Travellers to Low- and Middle-Income Countries: A Systematic Review

**DOI:** 10.3390/ijerph13121249

**Published:** 2016-12-16

**Authors:** Gemma Crawford, Roanna Lobo, Graham Brown, Chloe Macri, Hannah Smith, Bruce Maycock

**Affiliations:** 1 Collaboration for Evidence, Research and Impact in Public Health, School of Public Health, Curtin University, Perth 6845, Australia; roanna.lobo@curtin.edu.au (R.L.); graham.brown@latrobe.edu.au (G.B.); b.maycock@curtin.edu.au (B.M.); 2 Australian Research Centre in Sex Health and Society, La Trobe University, Melbourne 3083, Australia; 3 School of Public Health, Curtin University, Perth 6845, Australia; chloe.macri@graduate.curtin.edu.au (C.M.); hannah.j.smith3@graduate.curtin.edu.au (H.S.)

**Keywords:** expatriates, travelers, HIV, HIV acquisition overseas, sexual health, high- to low- and middle-income countries, population mobility

## Abstract

In some high-income countries, a proportion of human immunodeficiency virus (HIV), other blood-borne virus (BBV) or sexually transmitted infection (STI) diagnoses have been reported as acquired overseas in low- and middle-income countries. A review was conducted to explore HIV, other BBV or STI related knowledge, risk behavior and acquisition amongst expatriates and travelers, particularly males, travelling from high to low- and middle-income countries. Seven academic databases were searched for 26 peer reviewed articles that met inclusion criteria. Significant variability in the studies was noted, in age, travel duration and frequency and outcomes/risk factors measured and reported on. Risk factors described included longer duration of stay; being single; travel for romance or sex; alcohol and other drug use; lack of travel advice; being male; higher number of sexual partners; and inconsistent condom use. Vaccination, pre-travel health advice, and having fewer sexual partners were described as protective. Studies are needed focusing on the social context in which risk-taking occurs. Better collaboration is essential to deliver comprehensive health promotion interventions alongside more consistent pre- and post- travel testing and advice. Policy measures are crucial, including consistent evaluation indicators to assess impacts of HIV, other BBVs or STIs in the context of mobility. Risks and responses for these epidemics are shared globally.

## 1. Introduction

Population mobility is significant in scope, complexity and impact. It is an intrinsic feature of an increasingly globalized and borderless world [1,2]. Every year, more than three billion passengers travel by air [3] and over 50 million people travel from high to low- and middle-income countries [4,5]. Public health is confronted by issues inexorably linked to population mobility [6,7]. For example, evidence closely links population mobility with deleterious impacts on sexual health, including the transmission or acquisition of human immunodeficiency virus (HIV), other blood-borne viruses (BBVs) or sexually transmitted infections (STIs) [8]. Mobility has not only been identified as a driver of epidemics, it may also exacerbate existing risk factors, or increase individual vulnerability for acquisition of HIV, other BBVs or STIs [9]. This is influenced by push and pull factors, including motivation (employment, leisure) for, direction and destination (e.g., from the global south to the global north) of, and level of control (e.g., asylum, displacement) over travel [5].

HIV, other BBVs or STIs are some of the most commonly notifiable infections globally and are endemic in many low- and middle-income countries, particularly among priority populations (such as sex workers, men who have sex with men (via unprotected anal intercourse) or people who inject drugs) [10]. In 2012, the World Health Organization (WHO) estimated around 357 million new STI infections amongst those aged 15–49 years: trichomoniasis (*n* = 143 million), chlamydia (*n* = 131 million), gonorrhea (*n* = 78 million) and syphilis (*n* = 5.6 million) [10,11]. Additionally, as of 2015, there were an estimated 34.3 million people over the age of 15 years living with HIV [12]. The presence of an STI left untreated significantly increases the risk of acquisition and transmission of HIV [13]. The economic cost of STIs in the United States (U.S.) alone is around $16 billion (USD) in direct medical costs [14], notwithstanding the psychological and social consequences of STIs that have a major impact on quality of life [13].

During the last 25 years, population mobility has experienced significant growth both within and between countries and regions [1]. The United Nations World Tourism Organization suggested that for the first time, more than 1 billion people crossed international borders in 2012. Of those, one in two travelled for recreation or leisure and around a third for a range of reasons, such as visiting friends and family, or for religion or health care [15]. Amongst United Kingdom (UK) residents, international travel was common with an estimated 55 million visits overseas in 2010 [16]. In Australia, there were 16.9 million departures in 2014–2015, comprising 9.2 million Australian residents departing short-term, 7.3 million visitors and 391,200 permanent and long-term departures [17].

Increasingly permeable geopolitical borders quickly and easily link countries with high and low prevalence of HIV, other BBVs or STIs [18]. People migrate to high-income countries from low- and middle-income countries, and a growing number of people travel, constantly, semi-permanently or permanently from low prevalence, high-income countries, such as Australia and the UK, to regions where HIV, other BBVs and STIs are prevalent, particularly Sub-Saharan Africa and South East Asia. People may travel from high to low- and middle-income regions for purposes including working and volunteering, family reunion, leisure and tourism (including to seek sex), military and peacekeeping exercises, and retirement [19,20,21,22].

Travelling to and from countries of high HIV, other BBV or STI prevalence, places migrant and mobile populations at risk for communicable diseases [8] and enhances the likehood of onward transmission (particularly for those that do not know their infection status) in both the destination country as well as the country of origin (on redomestication). The context of risk is complex. The literature highlights a range of factors that may influence vulnerability for transmission and acquisition of HIV, other BBVs or STIs [23,24,25]. This includes frequency of travel to countries of high prevalence; participation in high risk sexual practices; use of protective behaviors (such as condoms); or, the presence of an untreated STI [26,27]. Further, risk may be mediated by knowledge of modes of transmission; access to health services (for testing, diagnosis and treatment); availability of travel advice; or the existence of supporting laws which do not criminalize practices of priority populations (such as men who have sex with men and sex workers) [28,29,30].

Increasing notifications have been observed, particularly of HIV, amongst migrants from low and middle-income countries travelling to high-income countries (acquired both prior to and after arrival in their destination country) [9]. Additionally, a number of high-income countries have reported increasing notifications of overseas acquired HIV, other BBVs or STIs, including those acquired in low- and middle-income countries [9]. Notwithstanding the inherent challenges in achieving the appropriate level of granularity in the way in which data is reported within and between countries, there is some merit in mentioning the broad trends that have been seen. For example, of UK-born adults diagnosed between 2002 and 2010, 15% (*n* = 2066) acquired HIV overseas, most commonly in Thailand, the U.S. and South Africa [31]. In Canada, from 2009 to 2011, 348 cases of blood-borne viruses or sexually transmitted infections related to travel were diagnosed via a CanTravNet site (of 3943 ill returned travelers) [32].

In Australia, HIV data are reported inconsistently across states and it has taken some time to harmonize surveillance data. Generally, surveillance data now shows where the individual was born and where HIV was likely acquired. Data from Western Australia show that between 2005 to 2009 and 2010 to 2014, the number of overseas acquired HIV cases increased by 56%. Of 731 new infections which were diagnosed in the period 2010–2015, 52% (*n* = 382) of cases reported overseas acquisition. Of these, a quarter (*n* = 93) were diagnosed amongst Australian born men, who had acquired HIV overseas most commonly in South East Asia or Sub-Saharan Africa [33]. In South Australia, 2014 data reported that 50% of cases had been acquired overseas (*n* = 28) [34]. In New South Wales (NSW), (the state with the highest HIV prevalence, historically acquired amongst men who have sex with men), 350 new diagnoses were made in 2015. Of these, 9% (*n* = 30) were born in Australia but likely acquired HIV overseas (compared with 6% of new diagnoses in 2009–2014). A further 19% (*n* = 65) were born overseas and likely acquired HIV overseas, compared with 15% of new diagnoses 2009–2014 [35].

Increasing travel to and from countries with high prevalence of HIV, other BBVs or STIs, coupled with contexts which may amplify risks, creates an emerging and important priority for public health [36]. Countries such as Australia have prioritized mobile and migrant populations in their national strategies for HIV, other BBVs or STIs [37]. Operationalizing this has proved more challenging, and addressing this issue presents challenges for clinicians, public health practitioners, policymakers and researchers to influence behavior and practices which occur beyond country borders [21]. Action is required to better understand the needs of both migrants and other mobile populations. Approaches and responses must be sensitive to culture and context and must not reify stigma towards specific countries or populations.

Few studies have been identified which examine the behaviors and contexts of HIV, other BBVs or STIs acquisitions amongst expatriates and travellers to low- and middle-income countries. However, the literature points to a number of opportunities for intervention and engagement such as pre-and post- travel advice, use of HIV treatments as prevention to reduce community viral load, in-country outreach and online and other health promotion interventions [9].

To explore this issue further, and as part of a larger study examining male expatriate and traveler social networks and risks for HIV and other STIs, we sought to build on previous reviews exploring traveller sexual health [20,22,28,30,38,39,40,41,42,43]. This work was undertaken concurrently with work examining the experiences, barriers and enablers related to HIV acquisition risk amongst migrants from low- and middle-income countries travelling to high-income countries. We reviewed existing evidence regarding the sexual health behaviors, experiences and outcomes (including HIV, other BBVs or STIs) amongst expatriates and travellers from high-income countries aged 18 years or older travelling to low- and middle-income countries. 

## 2. Materials and Methods

The review was conducted using the Preferred Reporting Items for Systematic Reviews and Meta-Analyses (PRISMA) guidelines [44]. Procedures used in this review followed those from other systematic reviews conducted by the Collaboration for Evidence, Research and Impact in Public Health [45,46,47]. The review was registered in the PROSPERO International Prospective Register of Systematic Reviews (Registration number: CRD42016033106). 

Only quantitative and qualitative primary studies, published in English, in peer reviewed journals, between the years 2000–2015 were included in the review. Studies included those conducted with: (1) adults (over the age of 18 years); (2) males; (3) expatriates and travelers; (4) from high-income countries travelling to low- and middle-income countries; and (5) exploring sexual health behaviors and harms. For the purpose of this review, high-income countries were those nominated as Organization for Economic Co-operation and Development (OECD) countries with a Gross National Income per capita above $12,746 (USD) [48].

The review excluded studies published prior to 2000 or after 2015; non-peer reviewed articles and grey literature; studies about participants under 18 years of age; studies specifically with women; studies on expatriates and travelers who move between high-income countries; studies on expatriates and travelers from low- and middle-income countries; and studies that did not focus on sexual health. Outcomes included demographics of travelers and individual characteristics; knowledge of sexual health behaviors; experiences; risk factors; testing and diagnosis of HIV, other BBVs or STIs. 

Seven databases were searched. Databases and search terms are listed in Table 1 below. All applicable variations, including Medical Subject Headings (MeSH) terms were used according to database specifications. PubMed, the Cochrane Library and Google Scholar were used to substantiate results of database searches. Reference lists from pertinent papers were examined to determine whether database results were exhaustive. Initial searched fields included keyword, title and abstract.

Endnote X7 (Clarivate Analytics, Philadelphia, PA, USA) citation management software was used to manage all articles. Two researchers conducted individual searches for each database, to ensure a full and comprehensive search was conducted with limited bias [49]. Articles from each database were imported into each researcher’s Endnote file. A search of the secondary databases was also conducted, using either the same search terms as the primary database searches, or key concepts, depending on the specificity of the database. Once all articles were imported, duplicates were removed. Endnote libraries were then combined and further duplicates were removed. Titles and available abstracts were screened for applicability based on inclusion criteria. Those not obviously relevant were removed. Citations were categorized into three groups for all databases: (1) possibly relevant studies; (2) background literature (including reviews); and (3) clearly irrelevant studies.

Only primary studies were included within the review. Reference lists of relevant literature and other reviews were manually searched to identify any other relevant primary articles as part of the inclusion assessment described above. Selected articles were reviewed in full where there was any uncertainty as to whether studies met inclusion criteria based on title and abstract alone. A quality appraisal was conducted to assess methodological quality of included studies. Two researchers conducted the assessment, using an adapted checklist [50,51,52]. The appraisal was then cross-checked by a further two members from the research team.

**Figure 1:**
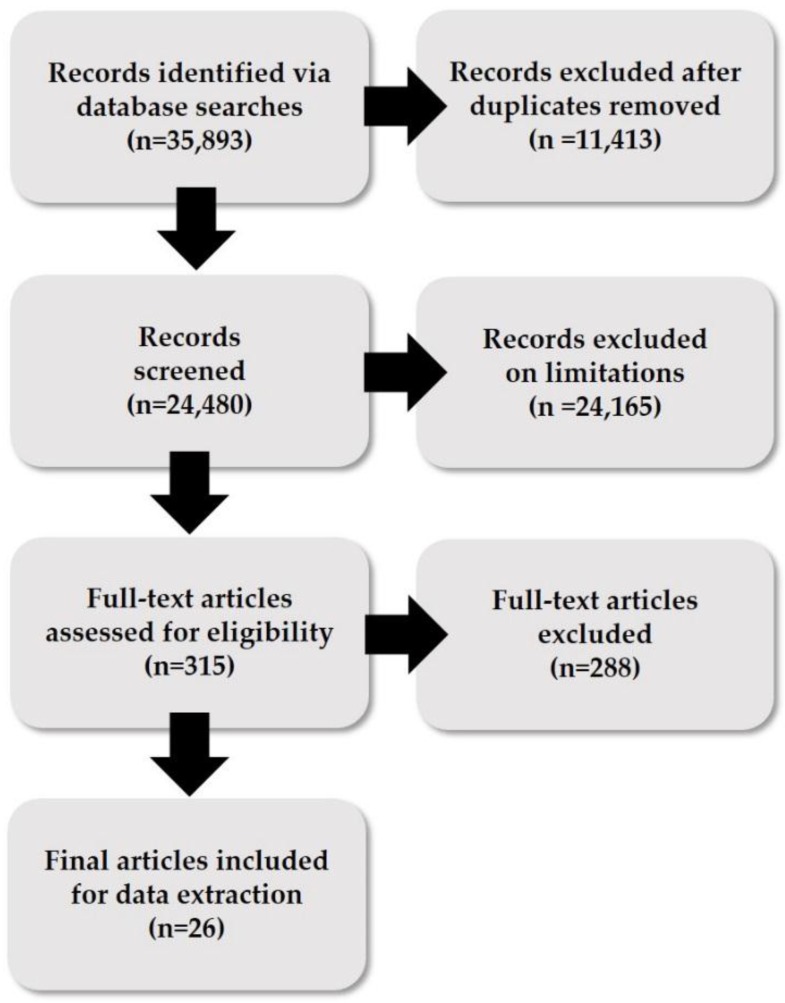
Flow diagram of review process.

A standard recording form was used to extract data of every study included in the review. This process was carried out by two researchers and then cross-checked by a second researcher to ensure consistency, facilitate accurate data presentation and confirm that there were no mistakes [49,53]. The data extracted included citation; participant characteristics; methods; results; and key conclusions of study authors. Figure 1 shows the process undertaken for the review.

## 3. Results

Twenty-six studies met the criteria for inclusion. The results have been categorized through the inclusion criteria, into multiple domains:

- Study Overview—design, setting, participant type and number
- Traveller and Travel Characteristics—gender, sexuality and age; participant country of origin and destination; purpose of travel, length of stay and frequency of travel
- Knowledge, Attitudes and Beliefs—perceptions of travel and destination countries; sexual expectations; knowledge of HIV, other BBVs or STIs
- Sexual Partner Acquisition—influencing factors; sexual mixing; commercial sex; number of sexual partners
- Alcohol and Other Drug Use—frequency of use, type, role as risk factor
- Condom Use—consistency of condom use; influencing factors
- Pre-Travel Health Consultation-use and experience; advice given
- Vaccination—knowledge and vaccination coverage
- Acquisition of HIV, other BBV or STI—risk behavior; diagnosis and related symptoms; place of acquisition; influencing factors
- Study Recommendations—policy, practice (clinical and health promotion) and research

A sample of the data extraction for the review and summary of key information extracted from the selected studies can be found in the Supplementary Materials (Table S1) attached to this review online and are reported for each included study under the following headings: author and purpose; origin and destination of travel; study details; sample and response; and reported outcomes.

### 3.1. Study Design and Setting

The final included articles comprised 21 quantitative studies [27,31,32,54,55,56,57,58,59,60,61,62,63,64,65,66,67,68,69,70,71] and five qualitative studies [29,72,73,74,75]. The size of the studies ranged from between eight and 34 participants in the qualitative studies and quantitative studies with up to 112,180 participants. The majority of the data collection for the studies occurred between 2000 and 2013. Four of the studies conducted consultations with patients during the 1990s, however all studies were published within the 2000 and 2015 date limits assigned for this review.

Of the 26 included studies, nine were conducted in Europe [55,59,60,63,64,67,68,71], five in the UK [27,31,56,65,69], four in each Australia [66,70,73,74] and South America [54,57,58,72], three in South-East Asia [29,62,75] and one in each Canada [32] and West Africa [61] respectively. Four studies [55,64,67,71] were conducted in travel medicine clinics and two South American studies were conducted in Peruvian airports [57,58].

### 3.2. Traveller and Travel Characteristics

Traveler and travel characteristics described are gender, sexuality and age; participant country of origin and destination; purpose of travel, length of stay and frequency of travel.

#### 3.2.1. Gender, Sexuality and Age

Twenty-two of the studies included both male and female participants; only four of the studies specifically targeted males. Sexuality was recorded in eleven studies [29,31,54,57,58,65,67,70,73,74,75]. Of these, six studies reported both heterosexual and homosexual participants [31,65,67,70,73,74], three studies reported heterosexual, homosexual and bisexual participants [54,57,58], while there was one study each that focused on homosexual participants only [75] and heterosexual participants only [29]. The studies varied in participant age with the majority including participants between 18 and 70 years.

#### 3.2.2. Origin

Specific country of origin was reported in 23 studies [27,29,31,32,54,56,57,58,59,60,61,62,63,65,66,67,68,69,70,72,73,74,75], with the majority of studies including participants from a range of countries. Amongst the 23 studies that reported participant origin, eight specifically originated in the U.S., seven were from Australia and six originated from England. Both the United Kingdom as a whole and The Netherlands had five studies each. Four of the studies originated from Germany, Sweden and general Europe respectively, while France and Belgium had three studies each and Italy had two. Austria, Switzerland, Spain, Finland, Canada, Scotland, Ireland and Japan were all represented once in the studies. 

#### 3.2.3. Destination

Destination of travel was reported in 23 studies [27,29,31,54,55,56,57,58,60,61,62,63,64,65,66,67,68,69,70,71,72,73,74]. Most of the studies included multiple destinations. The most frequently cited destinations were Asia broadly (*n* = 12), Central and South America (*n* = 10), Africa broadly (*n* = 9), Sub-Saharan Africa (*n* = 8), Thailand (*n* = 5), the Caribbean (*n* = 4), South-East Asia (*n* = 3), Peru specifically (*n* = 3) and North Africa (*n* = 2).

#### 3.2.4. Purpose of Travel

Purpose of travel was captured in 22 studies [27,29,32,54,55,56,57,58,59,60,61,62,64,66,67,68,69,71,72,73,74,75], with the majority of the studies reporting more than one purpose for travel. Tourism and vacation were most commonly reported (*n* = 17) followed by work and business (*n* = 12) and visiting relatives and friends (*n* = 7). Other less frequently reported reasons for travel included studying (*n* = 5), volunteering (*n* = 4), expatriation (*n* = 4), other (*n* = 4) and immigration (*n* = 1). Although most of the studies reported various reasons for travel, four of the studies specifically focused on longer term travel due to work [56,60,61,67] and three focused on lifestyle migration [73,74,75]. Nine of the studies recorded whether the travelers travelled with any partners or companions [54,57,58,59,60,62,63,71,72].

#### 3.2.5. Length of Stay and Frequency of Travel

Fifteen studies recorded length of stay of expatriates and travelers [27,29,32,54,55,56,57,58,59,60,61,62,64,66,67,71]. Stays ranged between six days and 11 months. The median length of stay reported for these studies was less than one month. For studies reporting on voluntary service overseas [56], aid work [60] and corporate expatriation [61], longer than six months of stay was most commonly reported. Five studies stated the frequency of travel of participants to particular destinations, ranging from a one-time occurrence, to more than eleven times in the past five years. [27,29,62,73,74].

### 3.3. Knowledge, Attitudes and Beliefs

Fourteen studies [29,57,58,59,62,63,65,66,68,69,72,73,74,75] reported on knowledge, attitudes and beliefs of travelers regarding sexual behavior and risk while overseas.

#### 3.3.1. Perceptions of Travel and Destination Countries

Five qualitative studies described perceptions of travel [29,72,73,74,75]. Studies by Brown and colleagues [73,74], Yokota [29] and Collins [75], reported specifically on perspectives and experiences among male travelers particularly travelling to Africa and Asia. The men within these studies often characterized their home countries as repressive, controlling and normative towards gender and sexuality. Factors identified that encouraged sexual activity and risk behaviors amongst men while overseas included perceptions that host countries are non-normative and that promote sexual freedom and participation in activities that are deemed “off-limits” at home [29,73,74,75]. Participants often sought out adventurous and different experiences, without an intention to assess the risks of doing so, or applied pre-existing understanding of risks in the home country to the destination country. Risk-taking behaviors such as the use of alcohol and other drugs, and multiple sexual partners were not uncommon [58,72,74]. These perceptions and attitudes toward mobility and sexual risk-taking behavior were shared among male Japanese travelers, particularly those seeking commercial sex in Thailand [29]. Knowledge of commercial sex services, specifically the known low-cost and wide availability of such services, were cited as contributing factors for increased likelihood of participating in commercial sex in Thailand [29]. Other attitudes identified towards mobility included a longing for a short-term escape or the sustaining of a new, long-term lifestyle within destination countries [73,74]. Several of the studies, including the study by Bauer [72] in Peru, suggested that engaging in sex overseas may offer a bridge for connection with the destination country’s culture, in order to successfully establish this long-term lifestyle, while mobility enables the opportunity for self-actualization and re-invention [72,73,74].

#### 3.3.2. Sexual Expectations

Nine studies [29,57,58,59,63,72,73,74,75] reported on attitudes concerning travelers’ expectations or intentions to have casual sex while overseas. Cabada et al. [57] identified travelers from the U.S. to have a greater expectation of casual sex in Peru and in turn, a greater number of sexual partners (*p* = 0.002), in comparison to those from Europe. Additionally, Croughs et al. [59] reported that the expectation to have sex overseas was more common among males in comparison to female travelers, with one-half and one-quarter expecting casual sex, respectively. The study by Manieri et al. [63] of Swedish male sex tourists in Thailand found that around half (48%; *n* = 76) of participants had expectations of casual sex with sex workers while overseas. Finally, Yokota [29] found perceived permissive norms toward commercial sex in Thailand which influenced expectations of casual or commercial sex.

#### 3.3.3. Knowledge of HIV, Other BBVs or STIs

Three studies reported on travelers’ knowledge of hepatitis B (HBV) and related risks [66,68,69]. Approximately half (45% [66] and 54% [69]) of travelers within two of these studies could define HBV or the related modes of transmission. The studies by Zuckerman and Hoet [68] and Zuckerman and Steffen [69] found however, a range of misconceptions including that hepatitis B was a result of excessive alcohol consumption, was rare as a sexually transmitted disease primarily affecting homosexuals and transmitted through contaminated food and water. Streeton and Zwar [66] determined that only one in five travelers identified HBV as a travel-related infection and only one in four knew of HBV vaccination availability. While one-third of travelers perceived themselves to be at risk for HBV exposure while in their home country (Australia), less than half (12%) of these travelers believed they were at risk of HBV exposure while travelling [66].

In relation to the risk of HIV exposure, Mercer et al. [65] reported greater perceived risk of HIV exposure among both women and men who had acquired a sexual partner overseas, compared to those who had not (*p* = 0.001). Manieri et al. [63] also reported a low (15%) mean risk estimate in relation to the perceived risk of HIV exposure through unprotected sex with a sex worker. Mercer et al. [65] also found that around 80% of respondents considered risks for HIV “somewhat more likely” or “much more likely” for individuals living in Thailand or Kenya. Brown et al. [73,74] found that men who identified as gay were more aware than those who identified as heterosexual, of preventative campaigns and testing to prevent HIV infection. Bauer [72] found generally poor levels of knowledge of STIs and safer sex within both locals and visitors to Cuzco associated with a lack of prevention campaigns and other education. Further, the study found that locals believed HIV infection was only associated with “homosexual behavior”.

### 3.4. Sexual Partner Acquisition

Nineteen studies reported on the acquisition of sexual partners while overseas [27,29,54,55,57,58,59,60,61,62,64,65,66,67,69,71,72,73,74]. Four studies purposefully sampled those either with an STI or those who had a sexual relationship overseas [29,64,71,74]. Of the remaining studies, the proportion of participants that had sex overseas ranged from 5% (*n* = 23 of 503) [66] to 52% (*n* = 245 of 468) [54], with between 5% and 30% most commonly reported.

Sex overseas was more frequently reported by male participants [27,57,58,59,60,62,67,69]. Other factors associated with greater likelihood of sexual partner acquisition and type included being single or travelling alone [29,57,58,59,60,65,67]; length of stay longer than one month [55,57,58,60,62]; premeditated expectation of sex overseas [58,59]; visiting multiple countries [67,68,69]; alcohol or other drug consumption [29,54,57,58,59,60,61,72,74]; identifying as homosexual or bisexual [57,58,65,67]; previous experience in the destination country [29,62], and a previous STI diagnosis [27,65].

#### 3.4.1. Sexual Mixing

Partner type was reported in 17 studies [29,31,54,55,56,57,58,59,61,62,65,67,71,72,73,74,75]. Locals in destination countries were the most commonly reported type of sexual partner (*n* = 15). Six of the studies reported between 42% and 67.3% of study participants engaged in sexual encounters with a local partner [55,57,59,62,67,71]. Of these, males were more likely than females to report a sexual encounter with a local companion (*p* < 0.05) [59,61,67]. Sexual encounters with other travelers were also documented, with Mercer et al. [65] reporting 50% of Britons having other UK nationals as their sexual partners, and 30% with partners travelling from European countries.

#### 3.4.2. Commercial Sex

Frequency of use, experience with or intention to purchase services of sex workers in the destination country was reported in 10 studies [29,31,54,57,58,62,63,73,74,75]. All Japanese male participants (*n* = 30) in the study by Yokota (2006) had engaged in commercial sex with a Thai sex worker in Bangkok, Thailand. Six studies [31,54,57,58,62,75] found only a relatively small proportion of participants had engaged sex workers. The proportion of participants that engaged sex workers in these studies ranged from 2%–25%. Participants in studies by Alcedo et al. [54], Cabada et al. [57,58] and Kaehler et al. [62] were more likely to report sex with other travelers or locals than sex workers. Rice et al. [31] reported that individuals acquiring HIV overseas were more likely to report purchasing sex (5%; *n* = 70 of 1516) than those who acquired HIV in the UK (0.7%; *n* = 51 of 7766) (*p* < 0.01). Further, engaging a sex worker was reported most frequently among men acquiring HIV in Thailand (11%; *n* = 39 of 347). Manieri et al. [63] reported on participant intention to pay for sex with Thai sex workers amongst men travelling for sex tourism (*n* = 158). Two-thirds of participants (63%) reported previous experience with sex workers, and around half (48%) had an intention to have sex with a sex worker during the current trip. While age was not associated with intent to engage a sex worker, relationship status (OR = 3.9) and prior experience with sex workers (OR = 17.7) were highly significant. No association was found between travel companionship and intent to purchase sexual services, though travelling alone (OR = 2.8) was somewhat significant. Both being single (OR = 5.0) and previous experience purchasing sex (OR = 43.3) were associated with an intent to purchase sex during the current travel.

#### 3.4.3. Number of Sexual Partners

Of the seven studies reporting the median number of sexual partners among travelers [54,57,58,60,67,71,72], one partner was most commonly reported [54,57,60,71], with the median number of sexual partners being three [54]. Male travelers were more likely than female travelers to report multiple sexual partner overseas [27,59,60,62,65,67]. The study by Whelan et al. [67] is one example of this; reporting that the median number of sexual partners among males was three, in comparison to female travelers who reported a median of two. Men who have sex with men and bisexual travelers were also more likely to acquire a sexual partner overseas, and at a more frequent rate in comparison to their heterosexual counterparts (OR = 6.17 (1.16 < OR < 33.5)) [57,58,65].

### 3.5. Alcohol and Other Drug Use

Nine studies reported on alcohol and other drug use while overseas [29,54,57,58,59,60,61,72,74]. Three studies specifically reported on alcohol use prior to sexual activity [54,57,58]. The proportion of participants that reported alcohol consumption prior to sexual activity ranged from 40% to 61.7%. Three studies specifically reported on other drug use prior to sexual activity with the proportion of participants ranging from 8% to 18% [54,57,58]. Croughs et al. [59] reported on the combined use of alcohol and other drug use, finding that around 80% of participants had used alcohol or other drugs prior to sexual activity. Women reported casual sex after using alcohol or other drugs more often than men (95% vs. 73%; *p* < 0.05) [59]. A further two studies reported generally on the use of alcohol or other drugs [60,61]. Dahlgren et al. [60] found that of 1029 participants, around 90% reported using alcohol overseas with 14% (*n* = 139) reporting an increase in use. The study found no association between time in destination country and increased alcohol use. Just under half (*n* = 14 of 32) of participants in the study by Hamer et al. [61] reported no change in alcohol consumption while in Western Ghana, however around a third (*n* = 11 of 32) reported increased consumption. Dahlgren et al. [60] found a small proportion of participants (2.9%; *n* = 34) reported using other drugs during their time in the destination country. Most reported cannabis use and were returning from Africa and Asia [60]. This was consistent with the study by Alcedo et al. [54] which found that of the 14.6% of participants using other drugs, cannabis was reported by around two-thirds.

Qualitative findings regarding the use of alcohol and other drugs while overseas ranged. Brown et al. [74] reported that alcohol was often perceived as part of a holiday or beach culture with prospects to “let your defences down with alcohol…”. The study by Bauer [72] suggested that alcohol may facilitate new contacts, while Yokota [29] also reported on the positive role that alcohol played in reducing social inhibitions. However, Bauer [72] also found that alcohol consumption could reduce inhibitions and impair judgement, facilitating unsafe sex.

### 3.6. Condom Use

Seventeen studies reported on condom use [29,54,55,56,57,58,59,60,61,62,63,66,67,71,72,73,74].

#### 3.6.1. Consistency of Condom Use

Studies reporting unprotected sex varied. More than half of participants within the studies reported on condom use, with consistent use ranging from 0%–87% [29,55,57,58,60,61,62,71]. In the study by Yokota [29], in Thailand, more than 85% (*n* = 26 of 30) of participants consistently used condoms with Thai partners, including sex workers. The lowest level of consistent use was reported by Ansart et al. [71] (*n* = 47).

Inconsistent condom use was reported in seven studies [54,56,57,59,67,71,72]. Alcedo et al. [54], for example reported three out of five participants using condoms inconsistently. Ansart et al. [71] and Whelan et al. [67] reported inconsistent use by two out of five participants, followed by Cabada et al. [57] and Croughs et al. [59] with approximately one in five and one in three (20% and 30.9%, respectively) of participants practicing inconsistent use. A number of studies also reported participants never using condoms [54,57,60,62,67,71,74]. For example, Ansart et al. [71] reported 60% of participants never using condoms, followed by 56% and 46% by Cabada et al. [57] and Whelan et al. [67], respectively.

#### 3.6.2. Factors Influencing Condom Use

Unprotected sex was reported as more likely to occur among travelers who were not in a relationship (*p* = 0.01) [67], and among those not receiving pre-travel health advice [59,61]. Inconsistent use did not differ between relationships with locals or other travelers [67]. However, Whelan et al. [67] also reported that for each additional partner acquired, the likelihood of sex being unsafe rose by 20%. Of those studies that reported participants who purchased sexual services from sex workers, Kaehler et al. [62] identified that two-thirds of these had consistently used condoms. In the survey by Manieri et al. [63] of Swedish men travelling to Thailand specifically for commercial sex, 20% had intended to practise inconsistent condom use, while 4% had intended to never use condoms. In a study of sex behavior of Japanese male tourists in Thailand, Yokota [29] found around 15% of participants practised inconsistent condom use with sex workers. While only one participant in the study by Cabada et al. [57] had engaged a sex worker, condoms had not been used.

Further reasons reported for not using condoms consistently reported in the qualitative studies by Bauer [72] and Brown et al. [73,74] included, that it was the “right feeling”; assumption that the “relationship was different”, consequently the “real thing”; trust and familiarity with partners; a sense of holiday romance; assessing a partner as a minor risk; being better not using them (“mas rico”); not deeming the sexual encounter to be “risky”, not wanting to interrupt “great sex”; throwing off the mantle of perceived previously “safe and cautious” behavior; sense of forming a new committed relationship; being portrayed negatively if carrying condoms and different perceptions of risk and patterns of risk behavior in home and destination countries [72,73,74]. A number of practical reasons were also cited for discontinuing use, such as cost, lack of knowledge and understanding, running out of condoms and embarrassment to purchase condoms [72].

A premeditated expectation to use condoms inconsistently also increased the likelihood of unsafe sex [59,62]. For example, although more than half of the participants in the study by Kaehler et al. [62], had condoms, only half of these (51.3%) intended to use them. Nevertheless, Croughs et al. [59] identified that individuals who carried condoms while travelling had a greater likelihood of safe sexual encounters than those who did not (OR = 5.4, 95% CI 1.7–17.0).

### 3.7. Pre-Travel Health Consultation

Use and experience of pre-travel health advice varied among ten studies measuring this [55,57,58,59,61,62,64,66,68,72]. Between 4% and 90% of participants within these studies had sought health advice prior to travel. For example, in the study by Zuckerman and Hoet [68] (*n* = 4151), around 90% of participants sought advice prior to travel. They found that more than half of participants sought this advice from a general practitioner and nearly 70% sought this advice five weeks or more prior to travel [68].

However, several studies found that up to half of participants had not received specific sexual health or hepatitis B information regarding risk factors and vaccination during their consultation [66,68]. Further, the Swedish travel clinic study by Angelin et al. [55] found that 113 participants reported advice received as irrelevant or inaccurate, with 14 participants reporting this specifically in relation to vaccination and vaccine preventable diseases.

In regards to gender and age, Angelin et al. [55] reported that males were less likely than females to seek pre-travel health advice (70% compared to 81%, respectively). Younger travelers benefitted less from pre-travel consultation in comparison to older travelers, and in turn showed a greater level of illness during travel and upon return (*p* < 0.001) [55]. In their study of U.S. and European travelers in Cuzco, Peru (*n* = 2540), Cabada et al. [58] found an association between pre-travel advice and casual sex whilst travelling (*n* = 77 of 997, *n* = 64 of 1539, OR = 1.92 (1.37 < OR < 2.71)). Further, they found that non-U.S. travelers received more pre-travel advice than those from the U.S. (*n* = 698 of 1587, *n* = 210 of 718, OR = 1.86 (1.54 < OR < 2.24)) [58]. This was consistent with findings from an earlier study by Cabada et al. [57] which also supported this statement (relative risk, 1.14; 95% CI 1.00–1.31). Additionally, Cabada et al. [57] showed that around 40% of participants received pre-travel advice with Canadian travelers having the highest frequency of pre-travel education (*n* = 23 of 33; 70%). Finally, Boggild et al. [32] found that among all ill returned non-immigrant travelers, the lowest levels of pre-travel advice were recorded amongst those travelling to visit relatives and friends (*p* < 0.001). 

### 3.8. Vaccination

Five studies reported on vaccination prior to overseas travel [59,66,67,68,69]. Reported coverage ranged from 17% to 74%. Croughs et al. [59] reported on vaccination amongst travelers from The Netherlands and Belgium who consulted a travel clinic prior to travelling (*n* = 1907). Forty-one percent of participants had not been vaccinated against hepatitis B, while 45% of participants had received at least one injection in relation to hepatitis B vaccination. Streeton and Zwar [66] also explored vaccination coverage with Australians travelling overseas (*n* = 503). While more than half of participants had travelled to a hepatitis B endemic region, less than half (43%) had been vaccinated either prior to their most recent trip or in a separate instance. Just under half (46%) of those exposed to at least one risk factor for hepatitis B during recent travel were not vaccinated. Those travelling to regions of medium to high hepatitis B endemicity had a greater likelihood of being vaccinated (52%; *n* = 146) compared to those travelling to low hepatitis B endemic regions (32%; *n* = 71) (*p* < 0.001). Travelers who were younger were more likely than older travelers to be vaccinated (58% of 18–29 year olds compared with 24% of those aged 50 years and older) (*p* < 0.001) [66].

Whelan et al. [67] conducted a study with long-term Dutch travelers (*n* = 552) to “sub- tropical” countries via pre- and post-travel surveys and pre- and post-travel blood sampling. Hepatitis B vaccination was offered to all participants prior to departure, of whom, 74% were fully vaccinated. Zuckerman and Hoet [68] and Zuckerman and Steffen [69] explored vaccination for hepatitis B via two separate telephone based cross-sectional surveys with participants from a range of European countries. In the first study, Zuckerman and Hoet [68] found that of 5948 participants, one in five had travelled to destinations of “moderate to high” hepatitis B endemicity in the past 5 years. Only 15% of 4151 travelers received vaccination against hepatitis B. A further one in five recalled being vaccinated for hepatitis but were unclear regarding the type they were vaccinated against. In the second study by Zuckerman and Steffen [69], with a cross sectional sample of 9008 participants, results showed that 17% (*n* = 1535) of travelers had been vaccinated for hepatitis B. One-quarter (*n* = 109) at high risk for hepatitis B had been vaccinated. 

### 3.9. Acquisition of HIV, Other BBVs or STIs

#### 3.9.1. Risk Behavior

Twenty-one studies reported on risk for HIV, other BBVs or STIs among participants travelling overseas [27,29,31,32,56,57,58,59,60,61,62,63,64,65,66,67,68,69,72,73,74]. The studies ranged from exploring participant awareness of the risks associated with HIV, other BBVs or STIs, as well as outcomes amongst participants who engaged in behaviors deemed to put them at risk for acquisition.

Eleven percent of volunteers (*n* = 24 of 215) in the study by Bhatta et al. [56] expressed concern that they had placed themselves at risk for HIV, other BBV or STI related symptoms, most commonly due to unprotected sexual intercourse. An association was also found between HIV, other BBV or STI risk behavior and age, where participants aged between 26 and 45 years had the greatest risk (*p* = 0.016). Kaehler et al. [62] identified that more than one-third (*n* = 10) of participants (*n* = 27) having sexual encounters with Thai sex workers were at risk of acquiring HIV or other STIs. In a study by Dahlgren et al. [60], approximately 12% (*n* = 41) of participants believed they undertook risky sexual behaviors they otherwise would not have taken at home. Furthermore, one in five participants (*n* = 68) reported taking a HIV test, while another 20% (*n* = 67) admitted to having a reason to take a HIV test due to risky behavior.

#### 3.9.2. Diagnosis and Related Symptoms

Ten studies reported on HIV, other BBV or STI diagnoses and related symptoms acquired during travel overseas [31,32,58,60,64,67,70,71,73,74]. The studies by Combs and Giele [70] and Rice et al. [31], examined retrospective epidemiological data of HIV cases within Western Australia and the UK, respectively finding 44% (*n* = 114 of 258) and 14% (*n* = 2066 of 13,891) of cases were diagnoses acquired overseas. Ansart et al. [71] and Matteelli et al. [64] reported specifically on STIs diagnosed at a travel clinic in Paris (*n* = 49 cases) and GeoSentinel clinics worldwide (*n* = 974 of 112,180). Genital gonorrhea, gonococcal urethritis, herpes simplex virus 2 (HSV2), syphilis (among those already diagnosed with HIV infection), chlamydia trachomatis, and HIV infection were identified as the most common STIs and BBV diagnosed among travelers presenting to those clinics. Similarly, in a study at the Canadian GeoSentinel sites of returned ill travelers (*n* = 3943), Boggild et al. [32] found 348 cases of BBVs and STIs including 15 cases of HIV infection. In the studies by Brown et al. [73,74], all participants (*n* = 14) had been diagnosed with HIV. In the study by Cabada et al. [58], 2.2% (*n* = 3 of 138) participants reported symptoms consistent with sexually transmitted infections during their time in Peru.

In the remaining studies, between zero and four diagnoses were reported. In a study of 219 returned overseas volunteers from the UK, Bhatta et al. [56] found that around 7.5% (*n* = 4) of participants had been diagnosed with an STI or BBV. In a similar study of returned Red Cross expatriates, Dahlgren et al. [60] found less than 1% had been diagnosed with HIV or other STIs or BBVs. This was despite being stationed in a country of high HIV prevalence and one in five returned expatriates (*n* = 67) reporting that they had reason to test for HIV. Finally, the study by Whelan et al. [67] with Dutch travelers (*n* = 552) found despite reported risk taking behavior, no participants had been diagnosed on their return home with HIV or other STI or BBV.

#### 3.9.3. Place of Acquisition

Place of acquisition was reported in seven studies [31,32,64,70,71,73,74]. Generally, Asia and Africa were the most commonly cited places of acquisition. For example, in the study by Combs and Giele [70], the majority of HIV acquired overseas amongst men was acquired in countries other than in their region of birth. South-east Asia was reported most frequently as the region of acquisition. This was consistent with the findings from studies by Brown et al. [73,74] and Rice et al. [31], which reported that amongst men who had sex with men, common locations of acquisition were U.S., Thailand and Spain.

#### 3.9.4. Influencing Factors

Contributing factors reported in the studies associated with HIV, other BBV or STI diagnoses included being male [64,70,71]; men who have sex with men [70,71]; travelling for non-tourist purposes [31,32,64]; or travel to a country of high HIV, other BBV or STI prevalence [31,64,70,71]. For example, Combs and Giele [70] reported males as being more than twice as likely (81%) as female travelers (29%) to acquire HIV overseas. This is consistent to studies reporting STI acquisition, with Matteelli et al. [64] reporting the likelihood of males and females acquiring an STI overseas as 67% and 33%, respectively. Pre-travel advice was found to be a protective factor in at least one study. Matteelli et al. [64] suggest that those who received health advice prior to travel were less likely to be diagnosed with an STI (0.5%) than those who had not (0.8%); this was statistically significant (*p* < 0.0001). 

### 3.10. Study Recommendations

All studies provided a range of recommendations for future policy, practice or research. Recommendations were generally related to public health or clinical practice, with all except four [21,24,57,69] of the studies making recommendations relating to travel health advice, education or health promotion. Around a third of the studies (*n* = 8) [21,51,55,57,58,64,67,68] provided recommendations for research including for behavioral or intervention design. Less than one in five of the studies (*n* = 5) [23,24,48,62,68] provided recommendations related to policy.

## 4. Discussion

### 4.1. Overview of Findings

This review aimed to (1) build on previous reviews exploring traveller sexual health; and (2) examine existing evidence regarding sexual health behaviors, experiences and outcomes (including HIV, other BBVs or STIs) for male expatriates and travellers aged 18 years or older from high-income countries travelling to low- and middle-income countries. In summary, we found 26 peer reviewed articles published between 2000 and 2015 that met the inclusion criteria. Table 2 summarizes the key results. 

Few protective factors were highlighted in this review. Those included were vaccinations and pre-travel health advice (particularly for older travelers), being female and fewer sexual partners [59,65,68], and perhaps participation in aid work. Risk factors highlighted in the review included destination, duration of stay and frequency of travel. A link was identified between travel to low-income destinations and an increase in risk taking behavior [63,70]. Travelers may seek sexual experiences and travel to destinations that are perceived to be less repressive, consequentially becoming less risk averse, especially males [29,63]. This was found to be more frequent in travel destinations such as South East Asia and South America [57,58,74]. Other findings report on the “situational disinhibition” that travelling itself presents, which can lead to increased risky behavior [28], suggesting there may be a relationship between length of stay or frequency of travel and disinhibition as people become more familiar and confidant with a location and its culture and environment. The relationship between travel destination and increase in risk taking behavior was also related to the duration and frequency of travel. A number of studies suggested that the longer participants were in the destination country, the greater the risk of having unprotected sex with a new partner [59,73], with a duration of stay over 30 days a key risk factor for unsafe sex while travelling [57,67].

Other risk factors included the number and type of sexual partners, condom use, alcohol and other drug use, gender and sexuality. Males were found to be generally at higher risk for acquisition of HIV, other BBVs or STIs [31,67,70] and had a great number of sexual partners overseas [67]. Men who have sex with men were also found to have an increased number of sexual partners [57,58], however only few included studies focused explicitly on this population. A number of the studies highlighted expectations of sex. Participants were often single and formed casual sexual partnerships while overseas [57,58,67]. This was found to be both unplanned and premeditative, with some travelers travelling specifically to destinations to seek sexual or romantic partners and/or sexual experiences [29,57,63]. Inconsistent condom use was found across studies with a range of barriers reported to their use including not deeming the encounter risky and not wanting to interrupt or take away from the encounter [72,73,74]. Unprotected sex was reported more frequently by travelers not in a relationship and those who did not receive pre-travel health advice [58,67]. Frequency, experience or intention to purchase or engage sex worker services in the destination country was reported in just under half of studies [29,31,54,57,58,62,63,73,74,75]. Arriving in the travel destination without a partner, participating in unprotected sex and having multiple sexual partners were all factors documented in other studies [22,38,76,77], as was the frequent purchasing of sex worker services and “sex tourism” industry overseas [30,78].

The review suggests that levels of knowledge and risks for transmission relating to HIV, other BBVs or STIs were poor [56,60,63,70]. Four studies suggested that alcohol and other drugs played a role in increased risk taking behavior, decreasing inhibitions and commonly used prior to sexual activity [54,57,58,59]. These results are consistent with findings from other studies, which highlight the relationship between alcohol and other drug use and sexual risk taking [20,76]. Finally, the reported pre-travel health advice among these studies was inconsistent and largely focused on health issues not specifically associated with sexual behaviors, such as malaria and parasitic infections [32,55]. Only four studies thoroughly discussed pre-travel advice for HIV, other BBVs or STIs [59,66,68,69]. The findings from this review are consistent with another systematic review regarding pre-travel advice [79]. The lack of advice specifically for HIV or other STIs and sexual health for travelers is apparent, with the review highlighting key recommendations for STI specific pre-travel advice [79].

Finally, while most studies included some recommendations, these mainly related to public health or clinical practice. Education and travel health advice were the key foci, for example prevention opportunities to increase vaccination rates [55,65,68,74]. Most failed to provide policy ready recommendations and only a third provided recommendations for research [29,57,58,61,64]. This is despite a number of previously completed reviews and policy documents [9], which provide explicit recommendations such as developing and increasing links and partnerships with affected communities, and creating closer cooperation with policy and support sectors in both origin and destination regions [9].

### 4.2. Study Design and Reporting Limitations

Papers included in this review cited a range of methodological limitations, with all studies bar two reporting limitations in research design, data collection or interpretation of results. More than half of included studies lacked reporting on ethics approval. Most included studies (*n* = 21) were quantitative. Of these, the majority were cross-sectional surveys which collected self-report data. Such methods may be disposed to measurement error which may weaken validity of findings [54,56,63,73]. The most frequent limitations outlined in the studies included over reliance on self-report measures; recall bias; variability in sample size and response rate, social desirability bias and self-selection bias. In addition, language barriers in data collection including lack of translated instruments, were highlighted in several studies [27,69].

Other limitations included the lack of standardized data collection instruments and lack of detail regarding validity and reliability of data collection instruments. Furthermore, few commonalities were found regarding the items within the instruments used to assess knowledge, attitudes, self-reported behaviors and outcomes. The studies included a range of ages and mixed gender samples which may have been limitations. Use of non-random, or non-representative samples were highlighted as limitations. There were few qualitative studies (*n* = 5) available to provide context to behavioral outcomes. For a number of the qualitative studies, there was a lack of in-depth analysis and reporting of findings against best practice reporting criteria [72,75]. Some studies also indicated a lack of in-depth interpretation using theoretical concepts or frameworks [72,75]. Overall studies used inconsistent definitions and categories (e.g., in relation to what constituted a sexual partner, relating to frequency of travel, traveler and expatriate). 

### 4.3. Strengths and Limitations of the Review

This review has a number of strengths. It provided a 15-year snapshot of the peer reviewed literature and built on previous reviews relating to HIV, other BBVs or STIs and travel from high to low- and middle-income countries. To our knowledge, it is the only study that sought to have an explicit focus on male expatriates and travelers, travelling from high to low- and middle-income countries. This has allowed an in-depth analysis of a particular priority population identified as requiring action and can assist the sector to support or refute a range of assumptions about the behavioral contexts in which acquisition of HIV, other BBVs or STIs occurs amongst expatriates and travelers to low- and middle-income countries. This may better guide policy and practice decision making and intervention design. 

The use of an established protocol used in other reviews provided a series of checks and balances. The use of seven databases provided expanded scope as it included a wide range of databases with multiple search terms and variations. To reduce any margin for error, multiple researchers conducted the database searching and a team approach was used to assess quality of the included studies. Including both qualitative and quantitative studies using a variety of methods expanded the scope of the review. The review was registered with the PROSPERO International Prospective Register of Systematic Reviews.

We recognize that there is a wealth of information available in the grey literature and within literature in languages other than English. Only including peer reviewed papers means that there have been an inherent level of publication bias. Most studies included both male and female participants, making it difficult to draw conclusions specific to the male expatriate and traveler population.

The inclusion of papers in languages other than English may have identified other relevant studies which may have enhanced the findings. We note that there are a number of countries that may have valuable experiences to contribute that may support or refute or provide additional context to our findings that do not have the resources to publish their findings in the peer reviewed literature.

Further, no meta-analysis or synthesis was conducted due to the heterogeneity and the high level of variability in the included studies. Thus consistent measures of quality were difficult to assess and we were limited in the conclusions that we are able to draw. Nevertheless, the study has updated the literature and addresses a gap in the literature regarding HIV, other BBVs or STIs and mobile populations. 

### 4.4. Implications for Research, Policy and Practice

There are a range of policy, practice and research implications from this review. Consistent with the principles outlined in the “HIV and Mobility in Australia: RoadMap for Action” [9], these incorporate international, national and local leadership and governance; community mobilization, enhanced service design and delivery; and ongoing surveillance, research and evaluation. These actions should be underpinned by a human rights approach that reduces barriers to testing and treatment, that commits ongoing resources, continues to resource effective strategies, and which acknowledges that addressing issues relating to mobile populations and the transmission and acquisition of HIV, other BBVs or STIs need more than information and education. Comprehensive, resourced and well evaluated strategies are required that do not demonize or penalize those most vulnerable [9]. The following sections outline opportunities to respond to and build on gaps identified in the included studies with reference to the broader literature.

#### 4.4.1. Research Opportunities

We found few studies that explicitly examined perspectives of migrants or expatriates and few which focused on men who have sex with men. Further, a number of the studies failed to segment target groups and included studies showed a lack of studies specifically focusing on men travelling to destinations of high prevalence. Consequentially, the studies provided broad findings that may not be relevant to the needs of those most at risk. For example, those who travel for extended periods of time or those who travel frequently may be at heightened risk, however may not perceive their risk to be high due to familiarity with the destination or because they believe that they are not part of a target group that is most at risk, such as holidaymakers or backpackers [29,73,74]. Accordingly, studies which better understand acquisition risks for different sub populations are required along with consistent data on destination, duration and frequency of travel. Most studies in this review were quantitative and cross-sectional and focused heavily on self-reported behavior and knowledge. It is important that valid standardized measures are incorporated and used to supplement self-report data provided by participants. This enables comparisons to be made across studies and results in the development of firm conclusions about current trends.

Few studies explored the settings in which risk taking behaviors occurred or the role of social or peer group influences. Historically, many high-income countries have viewed low- and middle-income countries through a colonial lens, created a perspective of them as permissive places, sources of infection or as playgrounds for those from more wealthy regions to engage in a range of behaviors that are viewed as less sanctioned in their country of origin [29,73,74,75]. There is a need for a greater level of contextual, qualitative social research which examines the diversity of perspectives of the target populations, particularly in relation to the perspectives of risk-taking and constructions of risk both in origin and destination countries [28,58,72,73]. Research that explores domestic attitudes, policies and practices which lead to risky behaviors among travelers is imperative [9,72,73,74,75]. Further, determining to what extent such laws and policies fuel negative attitudes in the general population towards low- and middle-income countries is vital. Further research may be valuable on social networks among long-term travelers to understand their function and role in sexual partnering and behaviors [9,36,73,74]. Studies which explore pathways and experiences of mobile populations, as individuals and as peer groups, may also better identify opportunities for policy and program intervention and for clinical practice [27,29,60,61,73,75]. Participation in aid work may provide a level of protection, however the studies describing this were inconsistent and further examination of this potential may be warranted, particularly to determine why risk behavior generally results in low levels of acquisition of HIV, other BBVs or STIs [56,60].

Based on the wide range of limitations in study design and type highlighted within the review, there is a need for methodological improvements for studies which are able to better inform the design and delivery of interventions as well as a greater level of applied intervention research. This may include identifying more specifically where HIV and other STI infections are occurring which will help target and tailor interventions, both in countries of origin and destination [9,32,58,71]. The review highlighted research opportunities which explore barriers and enablers to pre and post travel consultation and testing for both travelers and clinicians [32,55,57,66,68]. This could include intervention research to explore the efficacy and acceptability of treatment as prevention (such as pre-exposure prophylaxis for HIV) [64,69], for long-term travelers engaging in high risk behavior in destinations of high HIV, other BBV or STI prevalence [9,56,60,61,64,70].

#### 4.4.2. Clinical Practice Opportunities

This review highlighted implications that are important for clinical practice. A number of studies indicated poor knowledge and awareness regarding risks and protective behaviors related to HIV and other STI during travel [65,66,68,69,72,73]. This requires a reassessment of the role and scope of pre-travel counselling and advice, regarding sexual risk behaviors. Potential barriers should be addressed which prevent health professionals raising concerns with patients and instigating standard practice around recommending testing and treatment [31,56,58,68,69]. Guidelines for practitioners around pre-post travel consultation are needed as well as information and advice that is tailored to the context of travel [56,60,61,68,69]. Travel medicine providers should continue to provide information to travelers regarding HIV, other BBVs or STIs, but also undertake sexual health testing more regularly with travelers on return to countries of origin. Additionally, clinicians should consider the role of treatment in prevention for travelers at greater risk and provide consistent and systematic advice about carrying and using condoms [27,31,32,54,57,59,66,68,69]. Given the benefits of vaccination for hepatitis and the reported inconsistencies in knowledge, opportunities should be explored to increase vaccine coverage with follow up mechanisms, especially for older travelers [31,55,63,66,80].

#### 4.4.3. Health Promotion Opportunities

Recommendations for health promotion highlighted in the review mainly focused on the role of education [27,31,54,55,59,60,61,64,65,66,67,68,69,70]. Accordingly, there may be some scope to better deliver information or training to travelers as suggested in the review and as supported by the broader literature. This may be via traditional or new media (including smart-phone or other internet based tools) [55] or other suitable strategies to target specific mobile populations and travelers at greater risk [27,54,55,56,61], for example, those working in countries of high prevalence for protracted periods of time and males (and their partners), travelling to or through high prevalence countries [9]. This may include delivering in situ information in partnership with, or supportive of, local organizations [9,27,57,58,72,73]. Non-government organizations and employers could better engage with sex worker organizations at the local and regional level to better address risks for those seeking or engaging in commercial sex and reciprocal risk for sex workers [31,62,72,73]. Further consideration should be given to the development of partnerships with non-government and aid organizations working across borders and with transnational companies who employ people in countries of high prevalence and which experience significant cross border travel amongst their workforce as an opportunity for health promotion within the organization but also within the community more broadly [60,61].

A number of recommendations were made regarding working with airports, airlines and travel agencies as key points for information exchange or awareness raising [31,54,57,58]. However, to be most effective, multifaceted interventions including policy and environmental strategies as well as those tailored for individuals are likely to be most effective [9,31,64]. Segmentation of interventions which recognize heterogeneity of populations (e.g., recognizing key differences between older and younger travelers, men who buy sex and those that do not, men who have sex with men versus heterosexual men or those that are in situ for longer or shorter durations) may also be more effective, though cost benefits would need to be examined [9,55,65,67,69,71,72,73,80]. Finally, few studies discussed access to other preventive health measures such as safer sex or clean injecting equipment. Accordingly, there is a need to review the potential role for non-government organizations to trial strategies to increase access to and availability of equipment to reduce transmission risks [9,59,72].

#### 4.4.4. Policy and Advocacy Opportunities

Consideration should be given to the development of key indicators to evaluate programs for migrant and mobile populations which may assist to better identify what works and why [9,27,56,58,60,61,72,73]. Greater attention must be paid to both cross border and in-country responses. Ongoing monitoring and evaluation and harmonized surveillance is needed alongside more standardized jurisdictional surveillance for sub populations such as men who have sex with men [9,58,73,74,75]. Working with low- and middle-income countries to enhance their surveillance and publish their findings would increase our ability to effectively respond and reduce the impact of stigma and discrimination on specific populations and countries.

Despite being located in high prevalence regions for significant durations, the review found limited diagnoses amongst travelers, including volunteers and aid workers, despite a range of identified risk behavior. Protective and risk factors related to work roles should be further explored to determine the impact of workplace policies, education and access to equipment to reduce transmission risks, information and testing [56,60,61]. More advocacy and mobilization is needed from high-income countries to better support HIV, other BBV or STI testing (access and quality) within countries of high prevalence [9,31,60,73,74].

Countries such as Australia continue to enforce laws and policies that may have the consequence of making those most at risk more vulnerable for acquisition, including those relating to migration, sex work and drug use. Such laws and polices need to be re-examined in the context of cooperative cross border responses that recognize that HIV, other BBVs or STIs are co-transmitted. Where narrow protectionist policies (such as migrant screening on entry) exist, measures should be enacted to remove them [9].

## 5. Conclusions

Mobile and migrant populations are vulnerable for HIV, other BBV or STI acquisition, leading to significant health and other social impacts at the individual and community level. High-income countries have seen increasing acquisitions of overseas acquired HIV, other BBVs or STIs. Whist those travelling to and from countries with significant prevalence particularly of HIV have been identified as priority populations in a number of strategic frameworks, this review is one of few that has had an explicit focus on longer term travelers such as expatriates. The review revealed a high degree of heterogeneity among travelers and their behaviors, even when from similar sociodemographic backgrounds. This is a complex issue and one which requires greater inspection and a variety of tailored responses.

The domains identified in the 26 included studies included traveler and travel characteristics; knowledge, attitudes and beliefs; sexual partner acquisition; alcohol and other drug use; condom use; pre-travel health consultation; vaccination; acquisition of HIV, other BBVs or STIs; and study recommendations. This review found that the available evidence was limited in scope and inconsistent in study design and reporting. Accordingly, there is a need for future well-designed studies, particularly focusing on the social context in which risk-taking occurs. The review suggests that there are opportunities for public health to collaborate more closely with travel medicine and primary health care to deliver comprehensive multi-strategy health promotion interventions alongside more consistent pre- and post- travel testing and advice. Finally, well-funded and evaluated policy measures are needed as a matter of urgency, including advocacy for consistent evaluation indicators at a local, national and global level to assess the impact of HIV, other BBVs or STIs in the context of mobility.

HIV, other BBVs or STIs acquisition among mobile and migrant populations highlight that the risks and responses for these epidemics are shared globally. A “contain and control” approach or blunt migration strategy (that stops people at the borders) which has historically been used by high-income countries, abrogates their responsibility in relation to these epidemics. Policy and program responses in high-income countries which focus only on domestically acquired infections or conversely, only looking outward to destination nations to assume responsibility, will miss an important part of their epidemic as well as fail to meet reciprocal responsibilities to reduce cross border infections.

## Figures and Tables

**Table 1:** Search terms and databases used in the systematic review.

**Databases**	PsycINFO, MEDLINE, ProQuest, Scopus, Global Health, Web of Science, Embase
**Search Terms**	Sexual health related terms (“sexually transmitted infection” OR “sexually transmissible infection” OR “sexually transmitted disease” OR “sexually transmissible disease” OR “human immunodeficiency virus” OR “blood borne virus” OR STI OR HIV OR BBV OR STD OR sex OR “condom use” OR “sexual health” OR “sexual behavior” OR “sexual behavior” OR “sexual health risk” OR “sexual risk” OR “unsafe sex” OR “unprotected sex” OR “casual sex” OR “sexual intercourse” OR “sexual health behavior” OR “sexual health behavior” OR “venereal disease”)
	Expatriate and traveler related terms (expatriate OR traveler OR traveler OR “overseas volunteering” OR “military personnel” OR “aid work” OR “humanitarian aid” OR “lifestyle migration” OR “residential tourism” OR “international retirement migration” OR retirement OR retirees OR relocate OR relocation OR “transnational travel” OR “corporate travel” OR “business travel” OR “occupational travel” OR mining)
	Target group related terms (male OR men)

STI: sexually transmitted infection; HIV: human immunodeficiency virus; BBV: blood-borne virus; STD: sexually transmitted disease.

**Table 2:** Results Summary.

Overview	Twenty-six peer reviewed articles. Published between 2000 and 2015. High degree of variability in the study design and demographics.
Risk factors for acquisition of HIV or other STIs	Travel to a low-income region or region perceived to be less repressive, longer duration of stay.
	Single relationship status, travel specifically for romance or sex, (commercial or non-commercial).
	Alcohol and other drug use and not receiving pre-travel advice.
	Being male having a higher number of sexual partners and a lack of, or inconsistent condom use.
Other key findings in relation to HIV or other STIs	Levels of knowledge were poor.
	Few studies comprehensively discussed pre-travel advice.
Protective factors	Vaccinations and pre-travel health advice (particularly for older travelers).
	Being female and fewer sexual partners.
Recommendations	Lack of policy ready recommendations and only a third provided recommendations for research.
	Focus on education and travel health advice, for example prevention opportunities to increase vaccination rates.

## Supplementary Materials

The following is available online at www.mdpi.com/1660-4601/13/12/1249/s1, Table S1: Data Extraction Summary.
