# Supplementary material for: HIV, Other Blood-Borne Viruses and Sexually Transmitted Infections amongst Expatriates and Travellers to Low- and Middle-Income Countries: A Systematic Review
Source: Int J Environ Res Public Health. 2016 Dec 16;13(12):1249. doi: 10.3390/ijerph13121249 (PMC5201390; doi:10.3390/ijerph13121249)
Supplement: Supplementary file 1 [file ijerph-13-01249-s001.pdf]

Supplementary Materials: HIV, Other Blood-Borne Viruses and Sexually Transmitted Infections amongst Expatriates and Travellers to Low- and Middle-Income Countries: A Systematic Review

Gemma Crawford, Roanna Lobo, Graham Brown, Chloe Macri, Hannah Smith and Bruce Maycock

Table S1. Data Extraction Summary.

Author/Purpose	Origin/Destination of Travel	Study Details	Sample/Response	Reported Outcomes
**Alcedo et al. (2014) [1]**<br>To analyse factors associated with risky sexual behaviour among travellers	**Origin:** North America, Europe<br>**Destination:** Varied across North America, Africa, Latin America and/or the Caribbean, Europe, Asia, Oceania	**Design/Method:** Cross-sectional; online questionnaire.<br>**Participants/Recruitment:** Males and female; aged 18–35 years; recruited via Couchsurfing website.	**Sample:** n = 468<br>**Response rate:** 78%	• Sex during last travel<br>• Characteristics of sexual behavior<br>• Condom use
**Angelin et al. (2014) [2]**<br>To determine relevance of and adherence to health advice given to travellers to lower levels of travel-related illness	**Origin:** Sweden<br>**Destination:** Varied across Asia, Africa, South America	**Design/Method:** Prospective, cross-sectional; pre- and post-travel questionnaire.<br>**Participants/Recruitment:** Male and female; 18 years or older; Swedish speaking travelers attending a travel clinic.	**Sample:** n = 1277 (pre)<br>n = 1059 (post)<br>**Response rate:** 83%	• Perceptions of health advice<br>• Compliance with health advice<br>• Travel-related illness<br>• Risk behaviors while overseas
**Ansart et al. (2009) [3]**<br>To identify and evaluate STIs diagnosed among travellers consulting the health unit after returning from the tropics	**Origin:** France<br>**Destination:** Varied across America, Caribbean, Asia, Africa, Oceania	**Design/Method:** Cross-sectional; prospective; analysis of patient data.<br>**Participants/Recruitment:** Male and female; 18–49 years; returning travelers attending a travel clinic with signs of STIs.	**Sample:** n = 49<br>**Response rate:** 83%	• Signs indicative of STIs<br>• HIV status<br>• Sexual behavior<br>• Condom use
**Bauer (2007) [4]**<br>To explore tourists' and locals' knowledge, attitudes, and reasoning for engaging in casual sexual relationships	**Origin:** Varied across U.S., UK, Germany, Netherlands, Australia<br>**Destination:** Peru	**Design/Method:** Qualitative; in-depth, unstructured interviews; informal conversations; participant and non-participant observation.<br>**Participants/Recruitment:** Male and female; 19 years and older; locals linked to tourism or travelers for tourism, language courses or volunteer work recruited via convenience and snowball sampling.	**Sample:** n = 23<br>**Response rate:** Not Recorded	• Relationship type<br>• Sexual behavior<br>• Condom use<br>• Safe sex knowledge/education
**Bhatta et al. (2009) [5]**<br>To identify common health problems encountered by VSO volunteers during placement and after returning home	**Origin:** UK<br>**Destination:** Varied across North Africa, sub-Saharan Africa, Asia, Oceania, South America	**Design/Method:** Cross-sectional; self-complete post travel questionnaire.<br>**Participants/Recruitment:** Male and female; all ages; returned voluntary service overseas workers sent questionnaire and information pack on resettlement; completed anonymously, returned by mail.	**Sample:** n = 219<br>**Response rate:** 36%	• Demographics<br>• Pre-existing health conditions<br>• Illness suffered while on placement<br>• Illness upon return from volunteering

Table S1. *Cont.*

Author/Purpose	Origin/Destination of Travel	Study Details	Sample/Response	Reported Outcomes
**Boggild et al. (2014) [6]**<br>To identify the spectrum of illnesses experienced by Canadians travelling abroad	**Origin:** Canada<br>**Destination:** Varied across India, Mexico, Cuba, Dominican Republic, Costa Rica, U.S., Ghana, Thailand, Peru, China	**Design/Method:** Analysis of retrospective surveillance data of ill returned travelers from GeoSentinel database.<br>**Participants/Recruitment:** Male and female; all ages; returned travelers with probable/confirmed diagnoses, diagnosed at Canadian GeoSentinel clinics.	**Sample:** $n = 4365$<br>$n = 3943$ ill returned travelers<br>**Response rate:** Not Applicable	• Demographics<br>• Destinations of travel<br>• Travel purpose<br>• STI diagnosis<br>• Pre-travel health advice
**Brown et al. (2012) [7]**<br>**Brown et al. (2014) [8]**<br>To explore risk perspectives and experiences of Australian men who acquired HIV while travelling overseas	**Origin:** Australia<br>**Destination:** Varied across Asia, Africa, North America	**Design/Method:** Grounded Theory; semi structured interviews; symbolic interaction as theoretical perspective and analytical framework.<br>**Participants/Recruitment:** Males; 20 years and older; travelers who believed they had acquired HIV overseas between the years 2000–2009; recruited via through services accessed by people living with HIV, particularly AIDS Councils and hospitals. Majority of interviews face-to-face, but also online and telephone.	**Sample:** $n = 14$<br>**Response rate:** Not Recorded	• Destination, reason for travel<br>• Meaning ascribed to home and destination<br>• Knowledge of HIV<br>• Reported mode of HIV transmission<br>• Participant experience overseas-knowledge/attitudes/values
**Cabada et al. (2002) [9]**<br>To identify sexual behaviour and risk factors of travellers from the US and Europe to Peru	**Origin:** U.S., England, France<br>**Destination:** Peru	**Design/Method:** Cross-sectional; self-complete questionnaire.<br>**Recruitment:** Male and female travelers aged 15–51 years; departing from Peru on flights to the U.S. or Europe; convenience sampling in international departures lounge at airport.	**Sample:** $n = 442$<br>**Response rate:** 87%	• Sexual behavior<br>• Sexual expectations while travelling<br>• Condom use<br>• Sex partners while travelling
**Cabada et al. (2003) [10]**<br>To identify sexual behaviour and risk factors for STIs among travellers and locals interacting with travellers in Peru	**Origin:** U.S., England, France<br>**Destination:** Peru	**Design/Method:** Cross-sectional; self-complete questionnaire.<br>**Participants/Recruitment:** Male and female; 15–50 years; travellers; convenience sampling at airport and main bus stations prior to departure.	**Sample:** $n = 2540$<br>**Response rate:** 79.2%	• Demographics<br>• Sexual behavior<br>• Condom use<br>• Pre-travel health advice
**Collins et al. (2009) [11]**<br>To explore lived experiences of transnational mobility for gay-identified expatriates who reside in Manila	**Origin:** Varied across U.S., Great Britain, Germany, Scotland, Ireland, Sweden<br>**Destination:** Philippines	**Design/Method:** Ethnography; in-depth, informal field interviews.<br>**Participants/Recruitment:** male; gay; 29–70 years; expatriates; recruited at gay bars in Malate.	**Sample:** $n = 8$<br>**Response rate:** Not Recorded	• Experiences of gender, sexuality, nationality, race and mobility
**Combs and Giele (2009) [12]**<br>To analyse heterosexually acquired HIV cases observed among non-Aboriginal WA residents	**Origin:** Australia<br>**Destination:** Varied across Europe, Southeast Asia, sub-Saharan Africa	**Design/Method:** Descriptive, retrospective, cross sectional**;** analysis of Department of Health data of those newly diagnosed from 2002–2006.<br>**Participants/Recruitment:** Male and female; all ages; non-Aboriginal residents who had lived or intended to live in Western Australia.	**Sample:** $n = 258$<br>**Response rate:** Not Applicable	• Demographics<br>• Country of origin<br>• Reported place of HIV acquisition<br>• HIV exposure categories

Table S1. *Cont.*

Author/Purpose	Origin/Destination of Travel	Study Details	Sample/Response	Reported Outcomes
**Croughs et al. (2008) [13]**<br>To determine degree to which Dutch travellers receiving travel clinic pre-travel advice have protected or unprotected sexual contact with new partners and factors influencing this behaviour	**Origin:** Netherlands and Belgium<br>**Destination:** Varied across sub-Saharan Africa, Asia, Turkey, South America, Central America, North Africa	**Design/Method:** Cross-sectional; self-complete questionnaire.<br>**Participants/Recruitment:** Male and female; 18–50 years; travelers; Dutch speaking; questionnaire sent to travelers within 6 weeks of visiting a pre-travel clinic, followed by reminder.	**Sample:** *n* = 1907<br>**Response rate:** 55%	• Demographics<br>• Sexual behavior of travelers<br>• Condom use
**Dahlgren et al. (2009) [14]**<br>To assess self-reported health risk and risk-taking behaviours of humanitarian expatriates	**Origin:** Primarily Europe, America<br>**Destination:** Primarily Africa, Asia	**Design/Method:** Cross-sectional; self-administered questionnaire.<br>**Participants/Recruitment:** Male and female; all ages; humanitarian aid workers who had been on an ICRC mission for at least 1 month; contacted and asked to complete a questionnaire.	**Sample:** *n* = 1190<br>**Response rate:** 95.2%	• Demographics<br>• Health status<br>• Health related problems of workers<br>• Risk-taking behaviors
**Fenton et al. (2001) [15]**<br>To determine extent to which black African communities residing in London visit countries of birth, and the associated factors of acquiring new sexual partners while overseas	**Origin:** UK<br>**Destination:** Democratic Republic of Congo, Kenya, Uganda, Zambia, Zimbabwe	**Design/Method:** Cross-sectional self-complete questionnaire.<br>**Participants/Recruitment:** Male and females; all ages; from Sub-Saharan Africa residing in London; recruited at social and commercial venues, such as churches, universities, embassies, and bars in London using ethnically matched interviewers.	**Sample:** *n* = 756<br>**Response rate:** 75.6%	• Demographics<br>• Condom use<br>• previous diagnosis with ST<br>• Number of sex partners<br>• HIV testing<br>• Perceived peer group norms
**Hamer et al. (2008) [16]**<br>To evaluate use of pre-travel medical services, current knowledge, and behaviour among expatriate corporate workers stationed in Ghana	**Origin:** North America, UK, Europe, other high income countries<br>**Destination:** Western Ghana	**Design/Method:** Cross-sectional self-complete questionnaire.<br>**Participants/Recruitment:** Male and female; 21 years or older; corporate expatriate employees; field medical officer distributed questionnaire to all relevant expatriate employees.	**Sample:** *n* = 42<br>**Response rate:** 70%	• Demographics<br>• Pre-travel medical services<br>• Knowledge, behavior regarding a range of diseases and infections, alcohol use, high-risk sexual activity
**Kaehler et al. (2013) [17]**<br>To determine sexual behaviour and attitudes among foreign backpackers in Thailand	**Origin:** Europe, North America, Australia<br>**Destination:** Thailand	**Design/Method:** Cross-sectional self-complete questionnaire.<br>**Participants/Recruitment:** Male and female; 18 years and older; English-speaking backpackers without a spouse; using convenience sampling, participants approached in backpacker center in Bangkok.	**Sample:** *n* = 415<br>**Response rate:** Not Recorded	• Demographics<br>• Pre-travel preparations<br>• Sexual risk behaviors<br>• Condom use<br>• Selection of sex partners

Table S1. *Cont.*

Author/Purpose	Origin/Destination of Travel	Study Details	Sample/Response	Reported Outcomes
**Manieri et al. (2013) [18]**<br>To investigate sexual risk-behaviour of Swedish men who have sex with sex workers in Thailand	**Origin:** Sweden<br>**Destination:** Thailand	**Design/Method:** Cross-sectional self-administered questionnaire.<br>**Participants/Recruitment:** Male; all ages; Swedish citizens; recruited by male interviewers in the streets or inside bars and restaurants of the red-light districts of Pattaya and Bangkok.	**Sample:** n = 158<br>**Response rate:** 65%	• Demographics<br>• Experience with sex workers<br>• Intention to user Thai sex workers<br>• Condom use and perceived risk
**Matteelli et al. (2013) [19]**<br>To describe the range of diseases and factors associated with acquisition of travel-related STIs via the GeoSentinel database	**Origin:** Varied<br>**Destination:** Varied across Asia, Africa, America, America, Caribbean, Europe, Middle East, Oceania	**Design/Method:** Observational, cross-sectional; using standardized questionnaire to analyze diagnosed cases from GeoSentinel database.<br>**Participants/Recruitment:** Male and female; 13–90 years; crossed international borders within 10 years; confirmed/probable diagnoses	**Sample:** n = 112,180<br>**Response rate:** Not Applicable	• Demographics<br>• Travel history and reason for travel<br>• Pre-travel consultation<br>• STI diagnoses
**Mercer et al. (2007) [20]**<br>To determine the proportion of British residents who reported new sexual partners overseas in the past 5 years and the associated demographic, behavioural and attitudinal outcomes	**Origin:** UK<br>**Destination:** Varied across Europe, UK, Oceania, America, Caribbean, Asia, Middle-East, sub-Saharan Africa	**Design/Method:** Stratified national survey using multistage probability cluster design; face-to-face interviews using computer-assisted personal interviewing in respondents' homes, followed by computer-assisted self-interview.<br>**Participants/Recruitment:** Male and female; 16–44 years; travelers; British residents. A sample of addresses selected. For every selected household, one resident randomly selected to participate. Ethnic boost sample obtained with stratified postcode sampling.	**Sample:** n = 11,161<br>**Response rate:** main survey = 65.4%; ethnic boost sample = 63%	• Socio-demographics<br>• Health status, general risk factors<br>• Attitudes and knowledge of HIV<br>• Sexual attraction and experience<br>• Overseas travel<br>• Number of sex partners overseas<br>• Overseas sex partner demographics
**Rice et al. (2012) [21]**<br>To determine the characteristics of travellers born in the UK who acquire HIV infection overseas	**Origin:** UK<br>**Destination:** Spain, Nigeria, South Africa, Zimbabwe, USA, Jamaica, Thailand, other	**Design/Method:** Retrospective descriptive analysis; using case reports and follow-up data from national HIV database.<br>**Participants/Recruitment:** Male and female; 15 years and older; diagnosed with HIV infection in the UK; likely acquired HIV overseas.	**Sample:** n = 15,997<br>**Response rate:** Not Applicable	• Demographics<br>• Reported route of HIV transmission and country of infection
**Streeton and Zwar (2006) [22]**<br>To determine risk for hepatitis B exposure while travelling overseas for Australian travellers	**Origin:** Australia<br>**Destination:** Varied across Africa, Asia, Middle East, South and Central America, Europe, Oceania	**Design/Method:** Cross-sectional telephone survey.<br>**Participants/Recruitment:** Male and female; 18 years and older; had travelled overseas in the past two years, either for pleasure or business; recruited randomly via telephone calls to potential participants from each Australian mainland capital city using screening questions.	**Sample:** n = 503<br>**Response rate:** 74%	• Demographics<br>• Travel history<br>• Pre-travel health advice<br>• Uptake, adherence to pre-travel immunization<br>• Risks exposed to while travelling<br>• Perceptions, knowledge of hepatitis B

Table S1. *Cont.*

Author/Purpose	Origin/Destination of Travel	Study Details	Sample/Response	Reported Outcomes
**Whelan et al. (2013) [23]**<br>To determine the casual sexual relationships and condom use consistency among Dutch, long-term travellers to (sub) tropical regions	**Origin:** Primarily the Netherlands<br>**Destination:** sub-Saharan Africa, Central America, Caribbean, South America, Asia	**Design/Method:** Cross-sectional pre- and post-travel survey, pre- and post-travel blood sampling.<br>**Participants/Recruitment:** Male and female; 18 years and older; immunocompetent; travelers to (sub) tropical regions for at least 3–12 months; recruited via Public Health Service travel clinic.	**Sample:** n = 552<br>**Response rate:** Not Recorded	• Demographics<br>• Travel duration, destination, purpose<br>• Number, type, sex, ethnicity of sex partners<br>• Condom use<br>• HIV status
**Yokota (2006) [24]**<br>To explore reasons heterosexual male Japanese tourists engage in commercial sex in Thailand, and how motivations differ to those of Caucasian male tourists	**Origin:** Japan<br>**Destination:** Thailand	**Design/Method:** Qualitative semi-structured, in-depth interviews.<br>**Participants/Recruitment:** Male; 19–36 years; heterosexual; tourists; had sex with Thai sex worker(s) and who were travelling without partners; purposive sampling used to recruit participants in guesthouse lobbies.	**Sample:** n = 34<br>**Response rate:** 88%	• Demographics<br>• Sex with and history of commercial sex with Thai sex workers<br>• Condom use with Thai sex workers<br>• Reasons to buy sex from Thai sex workers
**Zuckerman and Steffen (2000) [25]**<br>To determine risks of hepatitis B infection among European travellers compared with immunisation status in other risk groups	**Origin:** Austria, Belgium, France, Germany, Italy, Netherlands, Sweden, Switzerland, UK<br>**Destination:** Varied-Primarily Africa, Asia, Central or South America	**Design/Method:** Cross sectional survey using telephone interviews with mostly closed questions (translated for all participants).<br>**Participants/Recruitment:** Male and female; 18 years and older; travelers; randomly sampled from telephone directories, using quotas.	**Sample:** n = 9008<br>**Response rate:** Not Recorded	• Demographics<br>• Travel destination (by endemicity)<br>• Risk behaviors<br>• Hepatitis vaccination status<br>• Knowledge
**Zuckerman and Hoet (2008) [26]**<br>To determine European travellers' risk for exposure and immunisation status of hepatitis B while travelling	**Origin:** Belgium, Italy, Finland, Germany, Netherlands, Spain, Sweden, UK<br>**Destination**: Varied across Africa, Asia, South America, Eastern Europe	**Design/Method:** Cross-sectional two-stage survey: (1) telephone Omnibus survey and (2) online survey.<br>**Participants/Recruitment:** Male and female; 18 years and older; travelers; to hepatitis B endemic countries; Omnibus survey participants chosen through random digit dialing used as quotas for participation in the online survey; Online survey participants recruited through online panel.	**Sample:** n = 5948 (Omnibus survey)<br>n = 4151 travelers (online survey)<br>**Response rate:** Not Recorded	• Demographics<br>• Travel frequency, purpose, destination<br>• Self-reported hepatitis B immunization status before travel<br>• Risk exposure to hepatitis B<br>• Pre-travel health advice

References

1. Alcedo, S.; Kossuth-Cabrejos, S.; Piscoya, A.; Mayta-Tristan, P. Factors associated with non-use of condoms in an online community of frequent travellers. *Travel Med. Infect. Dis.* **2014**, *12*, 750–756.
2. Angelin, M.; Evengard, B.; Palmgren, H. Travel health advice: Benefits, compliance, and outcome. *Scand. J. Infect. Dis.* **2014**, *46*, 447–453.
3. Ansart, S.; Hochedez, P.; Perez, L.; Bricaire, F.; Caumes, E. Sexually transmitted diseases diagnosed among travelers returning from the tropics. *J. Travel Med.* **2009**, *16*, 79–83.
4. Bauer, I. Understanding sexual relationships between tourists and locals in Cuzco/Peru. *Travel Med. Infect. Dis.* **2007**, *5*, 287–294.
5. Bhatta, P.; Simkhada, P.; Van Teijlingen, E.; Maybin, S. A questionnaire study of voluntary service overseas (VSO) volunteers: Health risk and problems encountered. *J. Travel Med.* **2009**, *16*, 332–337.
6. Boggild, A.K.; Geduld, J.; Libman, M.; Ward, B.J.; McCarthy, A.E.; Doyle, P.W.; Ghesquiere, W.; Vincelette, J.; Kuhn, S.; Freedman, D.O.; et al. Travel-acquired infections and illnesses in Canadians: Surveillance report from CanTravNet surveillance data, 2009–2011. *Open Med.* **2014**, *8*, e20–e32.
7. Brown, G.; Ellard, J.; Mooney-Somers, J.; Hildebrand, J.; Langdon, T. HIV risk among Australian men travelling overseas: Networks and context matter. *Cult. Health Sex.* **2012**, *14*, 677–690.
8. Brown, G.; Ellard, J.; Mooney-Somers, J.; Prestage, G.; Crawford, G.; Langdon, T. "Living a life less ordinary": Exploring the experiences of Australian men who have acquired HIV overseas. *Sex. Health* **2014**, *11*, 547–555.
9. Cabada, M.M.; Echevarria, J.I.; Seas, C.R.; Narvarte, G.; Samalvides, F.; Freedman, D.O.; Gotuzzo, E. Sexual behavior of international travelers visiting Peru. *J. Sex. Trans. Dis.* **2002**, *29*, 510–513.
10. Cabada, M.M.; Montoya, M.; Echevarria, J.I.; Verdonck, K.; Seas, C.; Gotuzzo, E. Sexual behavior in travelers visiting Cuzco. *J. Travel Med.* **2003**, *10*, 214–218.
11. Collins, D. "We're there and queer": Homonormative mobility and lived experience among gay expatriates in Manila. *Gend. Soc.* **2009**, *23*, 465–493.
12. Combs, B.C.; Giele, C.M. An increase in overseas acquired HIV infections among heterosexual people in Western Australia. *Sex. Health* **2009**, *6*, 35–39.
13. Croughs, M.; Van Gompel, A.; de Boer, E.; Van den Ende, J. Sexual risk behavior of travelers who consulted a pretravel clinic. *J. Travel Med.* **2008**, *15*, 6–12.
14. Dahlgren, A.L.; DeRoo, L.; Avril, J.; Bise, G.; Loutan, L. Health risks and risk-taking behaviors among International Committee of the Red Cross (ICRC) expatriates returning from humanitarian missions. *J. Travel Med.* **2009**, *16*, 382–390.
15. Fenton, K.A.; Chinouya, M.; Davidson, O.; Copas, A. HIV transmission risk among Sub-Saharan Africans in London travelling to their countries of origin. *AIDS* **2001**, *15*, 1442–1445.
16. Hamer, D.H.; Ruffing, R.; Callahan, M.V.; Lyons, S.H.; Abdullah, A.S. Knowledge and use of measures to reduce health risks by corporate expatriate employees in Western Ghana. *J. Travel Med.* **2008**, *15*, 237–242.
17. Kaehler, N.; Piyaphanee, W.; Kittitrakul, C.; Kyi, Y.P.; Adhikari, B.; Sibunruang, S.; Jearraksuwan, S.; Tangpukdee, N.; Silachamroon, U.; Tantawichien, T. Sexual behavior of foreign backpackers in the Khao San Road area, Bangkok. *Southeast Asian J. Trop. Med. Public Health* **2013**, *44*, 690–696.
18. Manieri, M.; Svensson, H.; Stafstrom, M. Sex tourist risk behaviour-an on-site survey among Swedish men buying sex in Thailand. *Scand. J. Public Health* **2013**, *41*, 392–397.
19. Matteelli, A.; Schlagenhauf, P.; Carvalho, A.C.; Weld, L.; Davis, X.M.; Wilder-Smith, A.; Barnett, E.D.; Parola, P.; Pandey, P.; Han, P.; et al. Travel-associated sexually transmitted infections: An observational cross-sectional study of the GeoSentinel surveillance database. *Lancet Infect. Dis.* **2013**, *13*, 205–213.
20. Mercer, C.H.; Fenton, K.A.; Wellings, K.; Copas, A.J.; Erens, B.; Johnson, A.M. Sex partner acquisition while overseas: Results from a British national probability survey. *Sex. Transm. Infect.* **2007**, *83*, 517–522.
21. Rice, B.; Gilbart, V.L.; Lawrence, J.; Smith, R.; Kall, M.; Delpech, V. Safe travels? HIV transmission among Britons travelling abroad. *HIV Med.* **2012**, *13*, 315–317.
22. Streeton, C.L.; Zwar, N. Risk of exposure to hepatitis B and other blood-borne viruses among Australians who travel abroad. *J. Travel Med.* **2006**, *13*, 345–350.
23. Whelan, J.; Belderok, S.; van den Hoek, A.; Sonder, G. Unprotected casual sex equally common with local and western partners among long-term Dutch travelers to (sub) tropical countries. *J. Sex. Transm. Dis.* **2013**, *40*, 797–800.

24. Yokota, F. Sex behaviour of male Japanese tourists in Bangkok, Thailand. *Cult. Health Sex.* **2006**, *8*, 115–131.
25. Zuckerman, J.N.; Steffen, R. Risks of hepatitis B in travelers as compared to immunization status. *J. Travel Med.* **2000**, *7*, 170–174.
26. Zuckerman, J.N.; Hoet, B. Hepatitis B immunisation in travellers: Poor risk perception and inadequate protection. *Travel Med. Infect. Dis.* **2008**, *6*, 315–320.

© 2016 by the authors; licensee MDPI, Basel, Switzerland. This article is an open access article distributed under the terms and conditions of the Creative Commons by Attribution (CC-BY) license (http://creativecommons.org/licenses/by/4.0/).
